# Prescription patterns demonstrate high demand for treating erectile dysfunction following radical prostatectomy

**DOI:** 10.2340/1651-226X.2025.42262

**Published:** 2025-03-02

**Authors:** Signe Benzon Larsen, Annika von Heymann, Hein V. Stroomberg, Anne Sofie Friberg, Klaus Brasso, Andreas Røder, Susanne Oksbjerg Dalton, Randi Karlsen, Pernille Envold Bidstrup, Annamaria Giraldi, Christoffer Johansen

**Affiliations:** a Department of Urology, Urological Research Unit, Copenhagen University Hospital – Rigshospitalet, Copenhagen, Denmark; b Cancer Survivorship, Danish Cancer Institute, Copenhagen, Denmark; c Section of Epidemiology, Department of Public Health, University of Copenhagen, Copenhagen, Denmark; d Danish Cancer Society National Cancer Survivorship and Late Effects Research Center (CASTLE), Department of Oncology, Copenhagen University Hospital – Rigshospitalet, Copenhagen Denmark; e Section of Biostatistics, Department of Public Health, University of Copenhagen, Copenhagen, Denmark; f Department of Clinical Medicine, University of Copenhagen, Copenhagen, Denmark; g Department of Clinical Oncology & Palliative Care, Zealand University Hospital, Naestved, Denmark; h Psychological Aspects of Cancer, Cancer Survivorship, Danish Cancer Institute, Copenhagen, Denmark; i Sexological Clinic, Mental Health Centre, Mental Health Services-Capital Region of Denmark, Copenhagen, Denmark

**Keywords:** Radical prostatectomy, erectile dysfunction, prescriptions, prostate cancer, epidemiology, clinical outcomes

## Abstract

**Background and purpose:**

Radical prostatectomy can cause erectile dysfunction; however, subsequent treatment with, e.g., phosphodiesterase-5 inhibitors may improve sexual function in the patients. We aim to examine prescriptions for erectile dysfunction after radical prostatectomy and to identify factors that may affect the prescription rate.

**Patients and methods:**

A study based on men included in the Danish Prostate Registry (DanProst) in 1995–2021, and information on prescriptions for erectile dysfunction (ATC: G04BE) from the Danish Prescription Registry. We calculated the proportion of prescriptions per month from 1 year before to 2 years after the initial biopsy and odds ratios (ORs) with 95% confidence intervals (CIs) for the risk of having a prescription.

**Results:**

We included 9,286 men with radical prostatectomy, 4,221 men managed on active surveillance, and 47,572 men with nonmalignant biopsies for comparison. The proportion of prescriptions increased significantly after biopsy among men with radical prostatectomy compared to men with nonmalignant biopsies and active surveillance. Patients with prior prescriptions for erectile dysfunction had an OR of 3.49 (95% CI, 2.98–4.08) of new prescriptions 6 months after the initial biopsy. Compared to patients treated with bilateral nerve-sparing surgery, patients with unilateral nerve-sparing surgery had an OR of 1.23 (95% CI, 1.06–1.43), whereas patients without nerve-sparing surgery had an OR of 0.40 (95% CI, 0.34–0.46).

**Interpretation:**

The observed patterns of prescriptions demonstrate a high demand for the treatment of erectile dysfunction following radical prostatectomy. The group of prostate cancer survivors is large, and, thus, a strong clinical focus on managing erectile dysfunction is needed.

## Introduction

A low mortality rate and a long expected survival following radical prostatectomy call for high attention to the quality of life within this group of cancer survivors [1]. Radical prostatectomy as a curatively intended treatment of localized prostate cancer is a well-established risk factor for erectile dysfunction affecting the quality of life [2–6]. In a British study of men with low-risk prostate cancer, a pretreatment erection firm enough for intercourse was reported by 67% of the men, but 6 months after treatment, the rate decreased to 12% among men treated with radical prostatectomy [5]. Throughout the follow-up, men treated with radical prostatectomy reported poorer sexual function compared to men either managed on active surveillance or treated with radiotherapy [7].

Drugs for erectile dysfunction can be used as prophylaxis and penile rehabilitation for patients undergoing radical prostatectomy [8–10]. A recent study among uro-oncology centers in Denmark, Finland, Norway, and Sweden found that all centers responding to the survey (*N* = 27) administered phosphodiesterase-5 inhibitors (PDE5 inhibitors) to men who had a nerve-sparing radical prostatectomy as an initial therapy [11]. Furthermore, a nationwide Swedish study of 25,390 prostate cancer patients showed that 74% of the patients treated with radical prostatectomy had filled at least one prescription for drugs used to treat erectile dysfunction 2 years after a prostate cancer diagnosis [12]. Preoperative erectile dysfunction is associated with an increased risk of erectile dysfunction after surgery [13]. Furthermore, the level of erectile dysfunction might be affected by conditions like obesity, depression, and metabolic disorders like hypertension and diabetes [14–18]. Among men with localized prostate cancer, the cancer-specific survival rates are almost identical among patients treated with active surveillance, radiotherapy, and radical prostatectomy [1]. However, the risk of complications after treatment differs significantly between the modalities, with the highest risk of bowel problems among patients treated with radiotherapy and the highest risk of urological symptoms and erectile dysfunction among patients treated with radical prostatectomy [7]. It is therefore important to consider a man’s medical history to assess the risk of erectile dysfunction and the man’s desire to maintain erectile function before the treatment strategy is determined.

A previous Danish study showed that the rate of prescriptions of drugs for erectile dysfunction increased after prostate cancer diagnosis among men with localized disease but not among men in the comparison cohort or among men diagnosed with non-localized disease [19]. This finding is likely caused by the major influence of radical prostatectomy, but the study lacked information on cancer treatment. The present nationwide study therefore elaborates on the rates of prescriptions of drugs for erectile dysfunction among prostate cancer patients treated with radical prostatectomy by comparing them to men with prostate cancer managed on active surveillance and men with nonmalignant biopsy findings. This study further investigates factors that may affect the proportion of prescriptions for erectile dysfunction among men treated with radical prostatectomy, including clinical factors related to prostate cancer diagnosis and previous prescriptions of selected commonly used.

## Materials and methods

We conducted a nationwide and population-based retrospective cohort study following the STROBE Guidelines [20].

### Data sources

Data were obtained from the Danish Prostate Registry (DanProst) containing information on all individuals with a histological examination of prostatic tissue in Denmark between 1995 and 2021, which currently include 190,422 men, of which 95,152 have been diagnosed with prostate cancer [21]. The time frame included the period from the first radical prostatectomy performed in Denmark to the latest update of the Danish Prostate Registry available.

By using the personal identification number (CPR) assigned to all Danish residents, we merged data from DanProst to obtain information on hospital contacts in the National Patient Registry, redeemed prescriptions in the Danish Prescription Register, and cancer diagnoses and clinical stage at the time of diagnosis in the Danish Cancer Register [22–24]. Data were extracted on September 3, 2024.

### Study population

**Figure 1:**
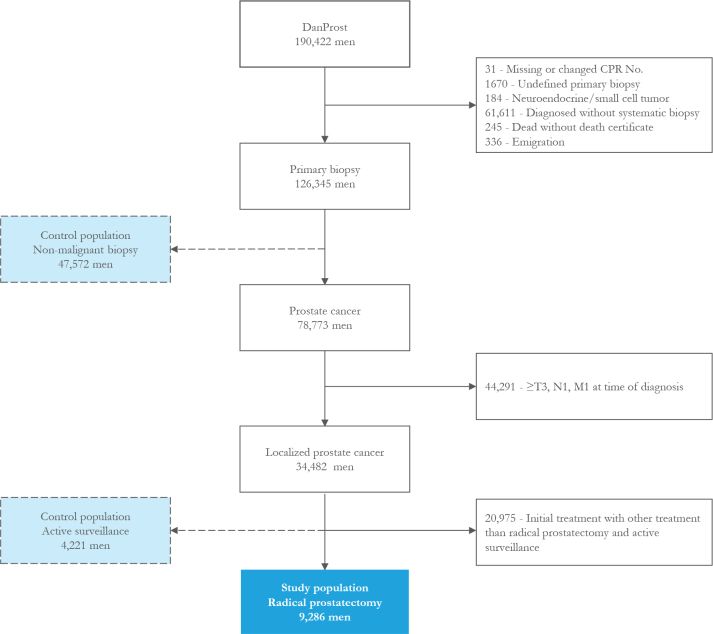
Flow chart.

The exclusion criteria were as follows: if registered with a missing or changed CPR number, undefined primary biopsy, neuroendocrine or small cell tumors, diagnosed without biopsy, died without a death certificate, and emigration (see Flowchart, Figure 1). The further excluding criteria are men diagnosed with advanced prostate cancer (≥T3, N1, or M1) and men with localized prostate cancer with other initial treatments than active surveillance and radical prostatectomy. Men with a nonmalignant primary biopsy were used as a non-cancer comparison group, and men managed on active surveillance were used as a comparison group with cancer to men treated with radical prostatectomy.

## Variables

Radical prostatectomy was stratified by type of operation (bilateral nerve-sparing, unilateral nerve-sparing, or not nerve-sparing) (see Supplemental Material for operation codes). From the Danish Prescription Registry, we obtained information on prescriptions for drugs used to treat erectile dysfunction (ATC group: G04BE, see Supplemental Material for details), hypertension (ATC: C02), diabetes (ATC: A10B), and depression (ATC: N06A). We further stratified drugs used to treat erectile dysfunction by injections (G04BE30 and G04BE01) and peroral administration (G04BE without G04BE30 and G04BE01). We included information on comorbidity as Quan’s updated Charlson comorbidity index (CCI), in which prostate cancer removed as a comorbidity (categorized into scores of 0, 1, or ≥2) [25]. D’Amico risk groups were categorized as low risk (prostate-specific antigen (PSA) < 10, Gleason score ≤6, ≤T2a), intermediate risk (PSA 10–20, Gleason score 7, T2b), and high risk (PSA ≥20, Gleason 8–10, ≥T2c) for men with sufficient information.

## Statistical analyses

The included men were followed from the date of initial biopsy until death, or end of follow-up on December 31, 2022. Characteristics at the time of the initial biopsy (age, comorbidity) or at the time of diagnosis (clinical characteristics) were reported descriptively for all men initially treated with radical prostatectomy. The prescription rate was defined as the number of prescriptions per 30 days divided by the number of men with full follow-up information within the same 30 days. The prescription rates were visualized for drugs related to the treatment of erectile dysfunction per month from 1 year before to 2 years after biopsy stratified by age at diagnosis, D’Amico risk group, nerve-sparing surgery, and comorbidity, as well as pre-diagnosis prescriptions for depression, hypertension, diabetes, and erectile dysfunction. Odds ratios (ORs) with 95% confidence intervals (CIs) were estimated to define the association of having a prescription for drugs used to treat erectile dysfunction 6 months, 1 year, and 2 years after the primary biopsy. The ORs were estimated by multivariable logistic regression adjusted for age and comorbidity. The ORs estimated among men treated with radical prostatectomy were further adjusted for D’Amico risk, nerve-sparing radical prostatectomy, and lymph node stage (N-stage). Estimates were considered statistically different if 1.00 were not included in the confidence interval. All statistical analyses were performed in R version (4.3.3).

## Results

The study population consisted of 9,286 patients with localized prostate cancer initially treated with radical prostatectomy compared to 47,572 men with a nonmalignant biopsy, and 4,221 patients initially treated with active surveillance (see flow chart in Figure 1). The median age at the time of biopsy was 64.7 years [interquartile range [IQR], 60.2, 68.6], and 97.5% had no comorbidities. Baseline characteristics of men treated with radical prostatectomy are shown in Table 1. Non-nerve-sparing radical prostatectomy was performed in 56.4% of the men, whereas 14.8% had double-sided nerve-sparing surgery. Positive lymph nodes were detected in 3.9% of the patients, and 39.5% had a lymph node dissection with no positive findings.

**Table 1:** Descriptive characteristics of 9,286 men treated with radical prostatectomy during 1995–2021 in the Danish Prostate Cancer Register.

Variable	Radical prostatectomy N = 9,286 n (%)
**Age**	
median [IQR]	64.7 [60.2, 68.6]
**Age groups**	
<65	4,842 (52.1)
65–70	2,899 (31.2)
>70	1,545 (16.6)
**Comorbidity**	
None	9,051 (97.5)
1	121 (1.3)
2+	114 (1.2)
**PSA**	
Median [IQR] (ng/mL)	7.7 [5.6, 11.4]
Missing	1,216
**PSA (ng/mL) grouped^a^**	
<4	489 (6.1)
4–10	5,076 (62.9)
10–20	1,894 (23.5)
>20	611 (7.6)
Missing	1216
**T stage**	
T1	2,354 (27.7)
T2	6,139 (72.3)
Missing	793
**N stage**	
N0	3,674 (39.6)
N1	362 (3.9)
Nx	5,250 (56.5)
**Gleason score**	
≤6	2,974 (32.0)
3 + 4	3,360 (36.2)
4 + 3 (5 + 2, 2 + 5)	1,172 (12.6)
7 unspec.	486 (5.2)
8	691 (7.4)
9–10	314 (3.4)
Undef AC	289 (3.1)
**D’Amico risk stratification**	
Low	2,031 (25.8)
Intermediate	1,678 (21.3)
High	4,153 (52.8)
Missing	1,424
**Nerve-sparing technique**	
Bilateral	1,376 (14.8)
Unilateral	2,672 (28.8)
No nerve-sparing	5,238 (56.4)

IQR, interquartile range; PSA, prostate-specific antigen.

a Men with missing PSA excluded.

**Figure 2:**
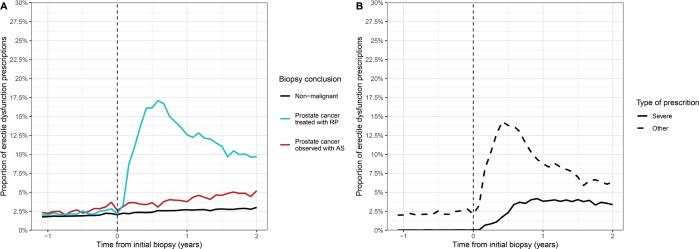
Proportion of prescriptions for erectile dysfunction before and after initial prostate biopsy stratified by (A) initial histological finding and treatment, and (B) type of prescription injections (G04BE30 and G04BE01) and peroral administration (G04BE without G04BE30 and G04BE01) of treatments for erectile dysfunction.

The proportion of prescriptions for erectile dysfunction increased rapidly within the first months after initial biopsy among men treated with radical prostatectomy, and a minor increase was observed among men managed with active surveillance compared to men with a nonmalignant biopsy (Figure 2A). The proportion of prescriptions for erectile dysfunction before biopsy was slightly higher among men subsequently managed with active surveillance compared to men with either subsequent nonmalignant biopsy or treatment with radical prostatectomy.

Compared to men with nonmalignant biopsy, the OR of a prescription among men managed with active surveillance was 1.5 (95% CI, 1.4–1.7) and 6.8 (95% CI, 6.4–7.1) among men treated with radical prostatectomy 6 months after biopsy (data not shown). After 1 year, the OR of a prescription for erectile dysfunction was 10.7 (95% CI, 10.2–11.3) among men treated with radical prostatectomy, whereas no change was observed for men managed with active surveillance. The ORs were similar 2 years after the biopsy.

When stratifying by type of prescriptions for erectile dysfunction, treatment with injections increased slightly from the time of biopsy but only to a limited extent and leveled out after approximately 6 months (Figure 2B).

**Figure 3:**
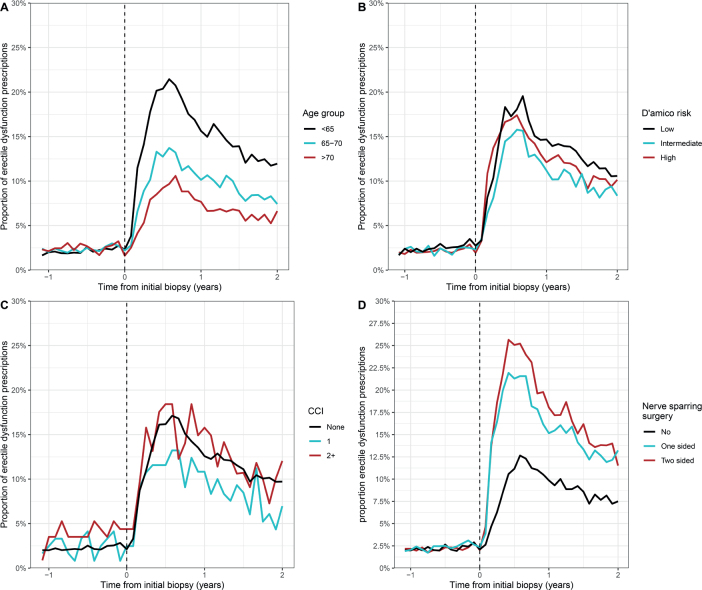
Proportion of prescriptions for erectile dysfunction among prostate cancer patients treated with radical prostatectomy before and after initial prostate biopsy, stratified by (A) age at the time of biopsy, (B) D’Amico risk, (C) comorbidity (CCI), and (D) type of surgery.

Among men treated with radical prostatectomy, the highest proportion of prescriptions for erectile dysfunction was observed among men younger than 65 years (Figure 3A). Neither D’Amico risk stratification nor the level of comorbidity affected the proportions significantly (Figures 3B and 3C). The proportions differed significantly between types of surgery, with the highest proportion observed among men treated with bilateral nerve-sparing surgery (Figure 3D).

**Figure 4:**
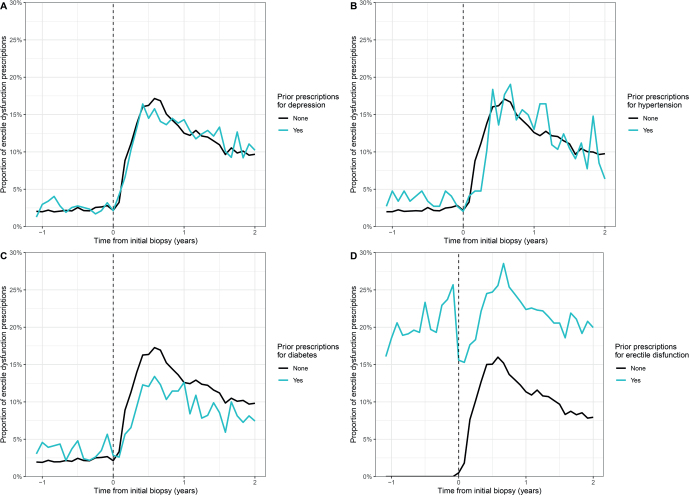
Proportion of prescriptions for erectile dysfunction among prostate cancer patients treated with radical prostatectomy before and after initial prostate biopsy, stratified by (A) prior prescriptions for depression, (B) prior prescriptions for hypertension, (C) prior prescriptions for diabetes, and (D) prior prescriptions for erectile dysfunction.

A prescription for antidepressants or antihypertensive treatment before biopsy did not affect the proportion of prescriptions for erectile dysfunction afterward (Figures 4A, 4B). Men with previous prescriptions for diabetes had a slightly higher proportion of prescriptions for erectile dysfunction before biopsy but a lower proportion after biopsy compared to men with no previous use of antidiabetics (Figure 4C). Among men with prescriptions for erectile dysfunction before the biopsy, the proportion of prescriptions dropped just before the biopsy but increased considerably again after the biopsy and stayed elevated, peaking at around 28% within the first year after the initial biopsy (Figure 4D).

The estimated ORs are shown in Table 2. After 6 months, the OR for prescriptions for erectile dysfunction was 0.67 (95% CI, 0.65–0.70) per 5 years increase in age, and the difference remained after 1 and 2 years. Among patients diagnosed with high D’Amico risk, the OR was 1.19 (95% CI, 1.05–1.34) 6 months after biopsy compared to those with low risk. There was, however, no difference after 1 and 2 years. Among patients with a CCI of 2+, the ORs were 0.59 (95% CI, 0.37–0.94) and 0.61 (95% CI, 0.38–0.98) 1 and 2 years after biopsy compared to patients without comorbidity, respectively. Among patients with previous prescriptions for diabetes, the OR was 0.65 (95% CI, 0.51–0.83) 6 months after biopsy and remained at that level after 1 and 2 years. Among patients with prescriptions for erectile dysfunction before biopsy, the OR was 3.49 (95% CI, 2.98–4.08) after 6 months and still elevated after 1 and 2 years. Compared to patients treated with bilateral nerve-sparing radical prostatectomy, the OR for prescriptions for erectile dysfunction was 0.40 (95% CI, 0.34–0.46) for patients treated without nerve-sparing surgery and 1.23 (95% CI, 1.06–1.43) for patients treated with unilateral nerve-sparing after 6 months. Six months after biopsy, there were no differences in the ORs for prescriptions between different lymph node stages; however, after 1 and 2 years, the OR was lower among patients diagnosed with N1 or Nx.

**Table 2:** Odds ratios (ORs) for prescription for drugs related to erectile dysfunction 6 months, 1 year, and 2 years after initial prostate biopsy in men treated with radical prostatectomy.

Variable	6 months		1 year		2 years	
	OR	95% CI	OR	95% CI	OR	95% CI
**Age**						
Per 5 years increase	0.67	0.65–0.70	0.63	0.60–0.66	0.60	0.58–0.63
**D’Amico risk stratification**						
Low	Ref		Ref		Ref	
Intermediate	0.89	0.77–1.04	0.88	0.76–1.02	0.87	0.74–1.01
High	1.19	1.05–1.34	0.98	0.87–1.11	0.93	0.82–1.06
**CCI**						
No	Ref		Ref		Ref	
1	0.76	0.48–1.19	0.87	0.58–1.33	0.91	0.59–1.39
2+	0.82	0.51–1.32	0.59	0.37–0.94	0.61	0.38–0.98
**Prior prescriptions for depression**						
No	Ref		Ref		Ref	
Yes	0.94	0.75–1.18	0.85	0.68–1.06	0.96	0.76–1.21
**Prior prescriptions for hypertension**						
No	Ref		Ref		Ref	
Yes	1.02	0.51–1.83	0.90	0.59–1.35	0.96	0.62–1.47
**Prior prescriptions for diabetes**						
No	Ref		Ref		Ref	
Yes	0.65	0.51–0.83	0.61	0.49–0.76	0.64	0.51–0.81
**Prior prescriptions for erectile dysfunction**						
No	Ref		Ref		Ref	
Yes	3.49	2.98–4.08	3.44	2.88–4.11	3.64	2.98–4.43
**Nerve-sparing**						
Bilateral	Ref		Ref		Ref	
Unilateral	1.23	1.06–1.43	1.14	0.96–1.35	1.15	0.96–1.38
No	0.40	0.34–0.46	0.39	0.34–0.46	0.42	0.35–0.50
**N stage**						
N0	Ref		Ref		Ref	
N1	0.99	0.76–1.30	0.82	0.64–1.05	0.85	0.66–1.10
Nx	0.94	0.84–1.04	0.87	0.78–0.97	0.87	0.78–0.98

OR, odds ratio; CI, confidence interval; CCI, Charlson comorbidity index

## Discussion

As expected, the proportion of redeemed prescriptions for drugs used to treat erectile dysfunction increased among men treated with radical prostatectomy compared to both men with a nonmalignant biopsy and with prostate cancer initially managed with active surveillance. The proportion of prescriptions increased slightly more among men managed on active surveillance than men with nonmalignant biopsy, which is likely caused by either more frequent contact with the healthcare system or subsequent progression leading to invasive treatment. It could, however, also be explained by a higher likelihood of being diagnosed with prostate cancer among men with erectile dysfunction, which could be indicated by the higher proportion of prescriptions for erectile dysfunction before biopsy observed among men managed with active surveillance. Among men treated with radical prostatectomy, lower age, bilateral nerve-sparing surgery, and redemption of prescriptions for erectile dysfunction before biopsy were associated with a higher proportion of prescriptions for erectile dysfunction. Higher age, non-nerve-sparing surgery, and prescriptions of antidiabetics before biopsy were associated with a lower proportion of prescriptions.

A Swedish study from 2014 found that the proportion of prescriptions for erectile dysfunction peaked at around 20% approximately 6 months after prostate cancer diagnosis among men treated with radical prostatectomy, which aligns with our finding of a peak of around 17%. [12]. The Swedish study further investigated the association between prescriptions and socioeconomic position and found that high income and long education were determinants of receiving drugs for erectile dysfunction [12]. The present study did not have access to socioeconomic variables; however, due to the comparability of the Swedish and Danish healthcare system, we expect the same tendency for socioeconomic position in Denmark.

The proportion of prescriptions for the treatment of erectile dysfunction with injections may indicate a stronger desire to maintain erectile function and showed a more limited increase compared to the more commonly used peroral drugs. The proportion of prescriptions for injections stayed stable throughout the follow-up, which indicates that either some men continue the treatment for a longer period or roughly the same number of men initiates and terminates the more severe treatment simultaneously.

We observed a lower proportion of prescriptions for erectile dysfunction among men with previous prescriptions of antidiabetics. It has previously been shown that men with diabetes have an approximately 3.5-fold higher prevalence of erectile dysfunction compared to men without diabetes [18]. The lower proportion is therefore most likely an expression of a lower expected effect of PDE5 inhibitors due to existing erectile problems. Although depression is also associated with erectile dysfunction, we did not observe any differences in prescriptions among men with and without prescriptions for depression before biopsy. A possible explanation could be that men with depression also have a high prevalence of low libido, which could affect the desire to maintain erectile function after cancer treatment [17].

The high quality of the nationwide Danish healthcare registries enables us to include many prostate cancer patients treated with radical prostatectomy, to compare with populations with and without a prostate cancer diagnosis, and to provide statistical power to stratify the analyses according to clinical factors, comorbidity, and previous prescriptions. The registries further secure an almost complete follow-up and the population-based design almost completely exclude social selection bias.

There are some limitations in using prescriptions to estimate erectile dysfunction. Due to the use of PDE5 inhibitors in prophylaxis and rehabilitation, the redemption of a prescription of drugs for erectile function is not equivalent to an existing condition [9]. Conversely, not having a prescription is not equivalent to not having erectile dysfunction but may indicate conditions where the patient is either unlikely to have any effect of the treatment or has no desire to maintain erectile function. The indication for the termination of treatment for erectile dysfunction is similarly difficult to establish since this can be caused by gain of function, limited effect, or lack of desire to maintain erectile function [5]. The findings of the present study should, therefore, not be interpreted as the prevalence of erectile dysfunction but, to a higher extent, the expectation of a positive effect of the treatment and the wish from the patient to maintain erectile dysfunction. Finally, Denmark has a welfare society providing financial support for prescription medicine, which may affect the proportion of prescriptions, and therefore the generalizability with other countries.

Prostate cancer is the most common male cancer diagnosis in the Western World, and men treated with radical prostatectomy have a low mortality, leading to a high number of urological problems including sexual dysfunction [26–29]. The patterns of prescriptions shown in this study clearly demonstrate a high demand for the treatment of erectile dysfunction following radical prostatectomy. Counseling of the patients regarding the choice of treatment is therefore important, and men with a high desire for the maintenance of erectile function may be better suited to receive either active surveillance or radiotherapy if possible.

In conclusion, the proportion of prescriptions for drugs used to treat erectile dysfunction was higher among men treated with radical prostatectomy compared to men with nonmalignant biopsy and prostate cancer patients initially treated with active surveillance. We further found that age, type of surgery, and previous prescriptions of drugs for erectile dysfunction were associated with a higher proportion of redeemed prescriptions for erectile dysfunction after biopsy. Overall, the prescription of PDE5 inhibitors for erectile dysfunction is commonly used after radical prostatectomy, indicating a high prevalence of the condition. Due to the large number of prostate cancer survivors, a strong clinical focus on managing erectile dysfunction is needed. The findings from this study provide knowledge regarding men at higher risk of prescriptions of PDE5 inhibitors that may be useful for future clinical interventions targeting erectile dysfunction after radical prostatectomy.

## Supplementary Material

Prescription patterns demonstrate high demand for treating erectile dysfunction following radical prostatectomy

## Data Availability

Access to data can be obtained by request to the corresponding authors and the Danish data protection authorities.
