# Supplementary material for: Prescription patterns demonstrate high demand for treating erectile dysfunction following radical prostatectomy
Source: Acta Oncol. 2025 Mar 2;64:42262. doi: 10.2340/1651-226X.2025.42262 (PMC11894291; doi:10.2340/1651-226X.2025.42262)
Supplement: Prescription patterns demonstrate high demand for treating erectile dysfunction following radical prostatectomy [file AO-64-42262-s1.pdf]

Supplementary material has been published as submitted. It has not been copyedited, or typeset by Acta Oncologica

Supplemental Table 1: NOMESCO classification of radical prostatectomies

NOMESCO	Type of surgery
KKEC00	Retropubic radical prostatectomy
KKEC00A	Retropubic not nerve-sparing radical prostatectomy
KKEC00B	Retropubic unilateral nerve-sparing radical prostatectomy
KKEC00C	Retropubic bilateral nerve-sparing radical prostatectomy
KKEC01	Percutaneous endoscopic radical prostatectomy
KKEC01A	Percutaneous endoscopic not nerve-sparing radical prostatectomy
KKEC01B	Percutaneous endoscopic unilateral nerve-sparing radical prostatectomy
KKEC01C	Percutaneous endoscopic bilateral nerve-sparing radical prostatectomy
**Grouping of surgery**	
KKEC00, KKEC00A, KKEC00B, KKEC00C, KKEC01, KKEC01A, KKEC01B, KKEC01C, KKEC10, KKEC20	All types of radical prostatectomy
KKEC00, KKEC00A, KKEC01, KKEC01A, KKEC10, KKEC20	Nerve-sparing radical prostatectomy
KKEC00B, KKEC00C, KKEC01B, KKEC01C	Not-nerve-sparing radical prostatectomy
KKEC00B, KKEC01B	Unilateral nerve-sparing radical prostatectomy
KKEC00C, KKEC01C	Bilateral nerve-sparing radical prostatectomy

Supplemental Table 2: ATC codes for drugs related to treatment of erectile dysfunction

ATC code		No of prescriptions	Percentage
G04BE01	Alprostadil	10,730	10
G04BE02	Papaverine	-	-
G04BE03	Sildenafil	57,826	55
G04BE04	Yohimbine	302	0.3
G04BE06	Moxisylyte	-	
G04BE08	Tadalafil	31,992	30
G04BE09	Vardenafil	2032	2
G04BE10	Avanafil	120	0.1
G04BE11	Udenafil	-	-
G04BE30	Combinations	2817	3
G04BE52	Papaverine, combinations	-	-
**Total no of prescriptions**		**105,699**	
